# Mitochondrial genome of the black-throated loon, *Gavia arctica* (Gaviiformes: Gaviidae): phylogeny and evolutionary history

**DOI:** 10.1080/23802359.2018.1473733

**Published:** 2018-05-15

**Authors:** Jae-I Moon, Jong-Gil Park, Sub Hur, Yeon-Kye Kim, Dong-Ha Nam, Dong-Hyun Lee

**Affiliations:** a Department of Biological Sciences, College of Natural Sciences, Chonnam National University, Gwangju, South Korea;; b Birds Research Center, Korea National Park Research Institute, Korea National Park Service, Jeollanam-do, South Korea;; c Food Safety and Processing Research Division, National Institute of Fisheries Science, Busan, South Korea

**Keywords:** *Gavia arctica*, Gaviidae, mitochondrial genome

## Abstract

We sequenced the complete mitochondrial (mt) genome of *Gavia arctica*. The circular mt genome is 17,065 bp long, consisting of 37 genes (13 proteins, 22 transfer RNAs, and two ribosomal RNAs) and a control region. Phylogenetic analysis based on the full mt genome sequences confirmed that the genus *Gavia* is a monophyletic group, containing the *G. stellata*, *G. arctica*, and *G. pacifica*. These data can provide insights into the phylogenetic relationships for inferring the pattern and degree of mt genome evolution among the loon species.

Specializations for foot-propelled diving have evolved independently in several avian species including loons (Gaviiformes: Gaviidae) and grebes (Podicipedidae); they are merely the outcome of convergent evolution in their hindlimb (Cracraft 1982). Loons are one of the oldest living lineages of birds that includes five extant species: *Gavia stellata*, *G. arctica*, *G. pacifica*, *G. immer*, and *G. adamsii* (Boertmann 1990). Among these species, black-throated loon (*G. arctica*) has an extremely wide range during breeding and wintering seasons across northern Europe, Asia, and coasts of the Atlantic and the Pacific (del Hoyo et al. 2014). However, the complete mitochondrial (mt) genome of this species has not been reported. Here, we sequenced the full mt genome of the *G. arctica*, which can help for its phylogenetic position and evolution of genomes.

During the wintering season, a *G. arctica* specimen was collected from the southern coast of Korea. We extracted the genomic DNA from the subsample (muscle) using the DNeasy Blood & Tissue kit (Qiagen, Valencia, CA) according to the manufacturer’s protocol, and the extracted DNA sample was deposited at the Wildlife Specimen Bank in Chonnam National University, Korea. We determined the complete mt genome sequence using the next-generation sequencing reads (450-bp length in each read) generated from MiSeq (Macrogen, Seoul, Korea). Mapped reads were used for *de novo* assembly and annotation by using commercial software (MITOS) to identify the full mt genome with about an average 150× coverage.

The complete mt genome of *G. arctica* was 17,065 bp in length (GenBank accession no. MH064399), and consisted of 13 protein-coding genes, 22 transfer RNA (tRNA) genes, two ribosomal RNA genes (srRNA and lrRNA), an origin of light strand replication site (O_L_), and a putative long noncoding control (D-loop) region. All three loon species (*G. stellata, G. arctica*, and *G. pacifica*) have the typical gene arrangement originally identified in the chicken. One protein-coding gene (NADH dehydrogenase subunit 6) and eight tRNA genes (*tRNA^Gln^*, *tRNA^Ala^*, *tRNA^Asn^*, *tRNA^Cys^*, *tRNA^Tyr^*, *tRNA^Ser^*, *tRNA^Pro^*, and *tRNA^Glu^*) were located on the light strand, whereas the rest of the genes (12 proteins, 14 tRNAs, and two rRNAs) were encoded on the heavy strand. The nucleotide composition of the *G. arctica* mt genome (A = 30.3%, C = 31.8%, G = 14.2%, and T = 23.7%) was similar to that of *G. stellata* (A = 30.4%, C = 31.4%, G = 14.4%, and T = 23.8%) and *G. pacifica* (A = 30.6%, C = 32.6%, G = 13.8%, and T = 23.0%). Comparisons between *G. arctica* and *G. pacifica* indicated a 95.1% sequence identity, and placed the *G. stellata* sister to these two species (Figure 1).

**Figure 1.**
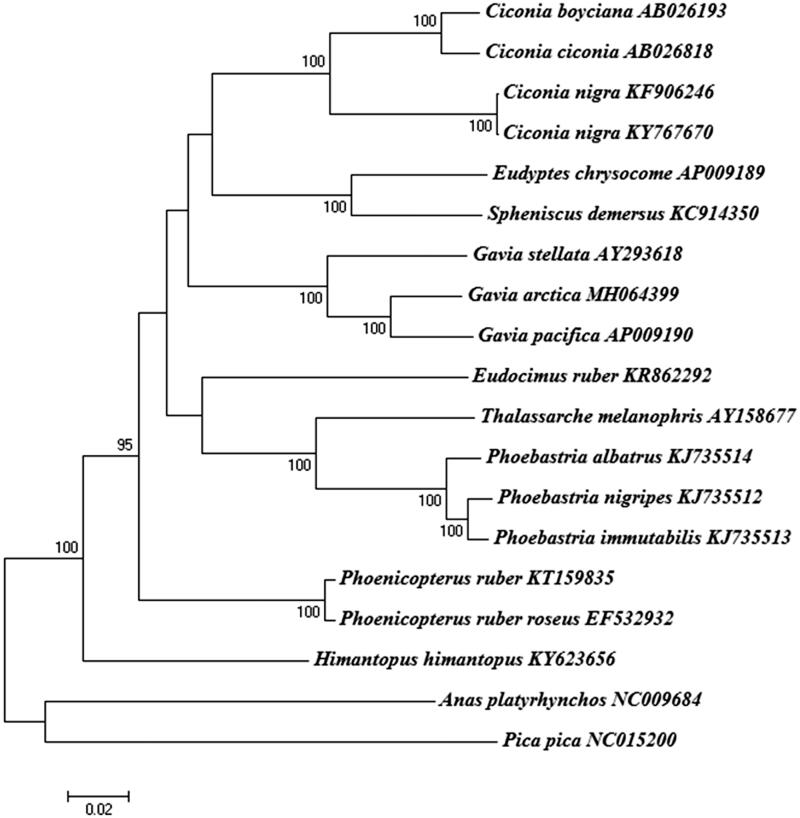
Phylogeny of *Gavia arctica* and other related species based on complete mitochondrial (mt) genome sequences. The complete mt genomes were downloaded from GenBank and the phylogenetic tree is constructed by a neighbour-joining method with 1000 bootstrap replicates containing the available full mt genomes. *Anas platyrhynchos* and *Pica pica* were used as outgroups for tree rooting. The percentage of replicate trees in which the associated taxa clustered together in the bootstrap test (1000 replicates) are shown next to the branches. GenBank accession numbers of each mt genome sequence are given in the bracket after the species name. The phylogenetic analysis was performed using MEGA7 to construct a neighbor-joining tree with 1000 bootstrap replicates (Saito and Nei 1987).

Phylogenetic analysis based on the full mt genome sequences revealed that *G. arctica* was clustered with genus *Gavia* (Family Gaviidae), showing a monophyletic group with two available loon species (Figure 1). A systematically close relationship has been reported among several taxa (e.g. Gaviiformes, Sphenisciformes, Procellariiformes, Podicipediformes, Ciconiiformes, and Pelecaniformes), but their phylogenetic relationships are still unclear (Watanabe et al. 2006). Our data indicated that loons (Gaviiformes) can share close relationships with Sphenisciformes and Ciconiiformes (Figure 1). These data provide insight into the phylogenetic relationships among the *Gavia* species and genomic evolution that might have played a role in speciation of foot-propelled divers.
